# Supportive hand-holding attenuates pupillary responses to stress in adult couples

**DOI:** 10.1371/journal.pone.0212703

**Published:** 2019-02-22

**Authors:** Tyler C. Graff, Steven G. Luke, Wendy C. Birmingham

**Affiliations:** Department of Psychology, Brigham Young University, Provo, Utah, United States of America; Groningen University, NETHERLANDS

## Abstract

**Background:**

Social relationships, particularly marriage, have been shown to ameliorate the potentially pathogenic impact of stressful events but prior research has been mostly aimed at downstream effects, with less research on real-time reactivity. Pupillometry is an innovative procedure that allows us to see the effects of acute stress in real time. The muscles that control pupil size are linked to the autonomic nervous system, so that when stressed, the pupils dilate; this occurs within 200ms. This quick response allows us to see the immediate effects of acute stress on the autonomic nervous system (ANS), and the real-time effects of social support in buffering stress.

**Purpose:**

The purpose of this study is to examine the dampening effects of received social support on the ANS’s pupillary response.

**Methods:**

Eighty individuals (40 couples) were randomly assigned to either a spousal support (i.e., spouse hand-holding) or non-support condition (i.e., alone) and administered a Stroop task while pupil dilation was measured.

**Results:**

The Stroop task elicited a stress reaction in terms of pupil dilation in response to the incongruent task trials. Participants in the support condition showed accelerated habituation to the stress task (*p <* .001), and less pupil reactivity (*p* < .001) providing evidence for buffering effects of social support via spousal presence and hand-holding.

**Conclusions:**

These results reveal the speed at which stress-buffering occurs, suggesting that pupillometry could be a good method to address the immediate dampening effects of social support.

## Introduction

The research linking supportive relationships with lower rates of morbidity and mortality is robust [1–3]. Supportive relationships are associated with better physiological and psychological health including immune and cardiovascular functioning, lower rates of depression, and better life satisfaction [4]. Additionally, lack of supportive relationships is associated with increased cardiovascular disease risk, depression, and poor immune function [5–7]. While social relationships may influence health via multiple pathways, one of the most widely researched of these is the stress-buffering model, which asserts that social relationships are primarily beneficial during periods of high stress and that these relationships can help ameliorate the potentially pathogenic effects of stressful events [8–12]. Stress is generally defined as “*a process in which environmental demands tax or exceed the adaptive capacity of an organism*, *resulting in psychological and biological changes that may place a person at risk for disease*.” ([13], p.3). Most definitions of stress maintain that stress contains key elements including (a) environmental, psychological, and biological phenomena, with an (b) emphasis on process, an (c) imbalance between environmental demands and adaptive capacity, and (d) playing a potential role in development and progression of disease processes. Stress can increase the risk of developing infectious disease, impact the severity of the disease, lower the strength of the immune response, and slow wound healing [14, 15]. Overwhelming evidence has linked stress to the development and progression of cardiovascular disease [16–18] with both acute and chronic stress linked to increased risk for morbidity and mortality from cardiovascular disease [17, 19, 20].

Stress can be acute (short-term) or chronic (long-term). Acute stressors can become chronic if one is exposed to the event repeatedly. Acute stress can include brief and prosaic events such as daily hassles or a relationship disagreement, or can be more impactful such as deadlines, illness, a hostile work interaction [21], or perceptions of social evaluation [22]. Importantly, social support has been shown to moderate the effects of both acute and chronic stress.

Social support can be the actual provision of support in times of need (received support; e.g. emotional, tangible, informational), or the perception that support is available if needed (perceived support). These two dimensions of social support are distinct constructs based on assumptions of different antecedent processes between them [23]. That is, received support is a situational factor that arises in response to stressful circumstances, whereas perceived support is not situationally-dependent [24, 25]. For example, when faced with acute stressful situations, received support could be simply the presence or close proximity of a friend or loved one, which can reduce psychological distress [26–29]. Social Baseline Theory [30] asserts that close physical proximity to a close other unconsciously guides perception and appraisal of situations through shared joint attention, goal sharing, and load sharing. The absence of a close other results in tasks being more cognitively, emotionally, and physiologically effortful, and perceived as more stressful [31]. Beyond the simple presence of a loved one, physical contact such as hand-holding has been shown to reduce anxiety associated with a stressful event [32, 33]. In his review of emotional support, Burleson [34] noted that emotional support can include non-verbal communicative behaviors (e.g., hugs, hand-holding, and focused looks) that are enacted with the intent to help another cope. Emotional support conveyed through hand-holding elicits clear, non-verbal communication of comfort and security [34, 35]. Recent research has shown that simply imagined touch support, such as hand-holding, has stress-buffering benefits [36]. Coan et al [33] found that emotional support in the form of hand-holding decreased neural responses to threat. Holding the hand of one’s spouse decreased the threat response even more. This could be because for most adults, their relationship with their spouse is the most important relationship in their life [37] and spouses are often the primary source of emotional support [38].

Although much has been learned about the mechanisms by which social support impacts health (e.g., cardiovascular, endocrine functioning, and immune system; see Robles and Kiecolt-Glaser [39] for a review), there are yet unexplored processes that could provide further understanding. Cardiovascular, endocrine, and immune reactivity is not captured instantaneously. The time frame of each method offers a different picture to understanding stress-buffering. Immune responses can take hours or even days [40]. Endocrine stress response onset can start as early as 15 minutes after an acute stressor [41, 42]. Cardiovascular measures are faster, usually occurring in minutes or even seconds. However, pupillometry is a method that can more instantaneously (milliseconds) capture the physiological response of stress. An examination of the body’s *immediate* physiological stress reactivity may help more fully understand the impact of acute stress and the buffering effect of social support on acute stress responses. For example, an immediate response to acute stress could be informative when assessing couples’ relationships and identifying specific problem areas within the relationship. Similarly, this immediate response could be useful in interventions where there may be several contributing variables of interest with varying effects. Additionally, pupillometry adds to the growing literature by providing another method to assess acute physiological stress response. In this way, the commonality of several measures could converge allowing investigators to place more confidence in the findings from diverse methods [43], thus more robustly examining and understanding this concept. Researchers have called for further work in order to better understand the causal mechanisms linking relationships to better health [44–47].

### Pupillometry

The autonomic nervous system (ANS) regulates important bodily activities including blood pressure. It includes both the parasympathetic nervous system (PSNS) and the sympathetic nervous system (SNS), which is responsible for reacting to threats and enhances the fight-or-flight reaction [48]. Well-known indicators of stress reactivity include galvanized skin response [49, 50], blood pressure and heart rate increases [51], decreased immune response [52], and hormone elevation [53]. Additionally, when individuals are under stress, the pupils enlarge [54].

The primary influence on pupil dilation is a change in luminance. However, smaller pupil dilations, usually less than 0.5 millimeters [55] also occur in response to psychological stimuli. These changes are controlled primarily by the locus coeruleus (LC) [56] which plays a significant role in autonomic activity generally; increases in LC activity lead to heightened sympathetic arousal, while decreases in LC activity result in activation of the parasympathetic system (see Samuels and Szabadi [57] for a review). Specifically, the LC responds to stress by increasing norepinephrine secretion through the hypothalamic–pituitary–adrenal (HPA) axis. Importantly, because the LC influences pupil dilation, the measurement of pupil size (pupillometry) is a method of assessing immediate, real-time stress response and recovery. The pupils begin to dilate within 200ms in response to the onset of stressors, as well as to increased cognitive or emotional arousal [58]. Once the stimulus is no longer attracting attention, because it has been recognized, removed, responded to or habituated to, the pupil size returns to baseline within a few seconds [59]. The time it takes for the pupil to return to baseline is a function of the stimulus presented: the more cognitive arousal, the longer the recovery [58].

Ren, Barreto, Huang et al [60] showed that pupil dilation measurement is a promising avenue for detecting stress and that instrument reliability is comparable with traditional methods such as galvanized skin response. Additionally, pupil dilation and constriction are unconscious and involuntary; one cannot control or suppress the pupil’s physiological response to stressors, making this method a good measurement of stress that may otherwise not be fully recognized by the individual. Indeed, pupillometry has been a validated form of measuring ANS activity and stress [54, 61–65]. Thus, pupillometry is a promising additional method of investigating the physiological stress-buffering effects of social support.

To test our research question, the present study used a Stroop task. The Stroop task is a word incongruence task that tests mental (attentional) vitality and flexibility [66]. A word is displayed in a color different from the color it actually names; for example, the word *blue* is presented in green font. In this task, participants must name the color of the font and inhibit their initial reactions to read the word. The Stroop task has been widely used in concert with pupillometry with research confirming that naming incongruous color-words evoked significantly greater pupillary dilation than congruent color-words [59, 60, 67, 68]. The Stroop task has also been widely used as a psychological stressor [49, 64, 69–72]. Boutcher and Boutcher [70] showed that performance on the traditional Stroop task (verbal response) caused a significant physiological stress response that manifest in cardiovascular, forearm blood flow, and epinephrine reactivity above that of the Stroop task without verbal response. This stress effect is due to visual attention being influenced by linguistic processing [73].

As shown in previous research, the stress-buffering effect, inherent in spousal relationships, may dampen the body’s physiological response to stressful stimuli [4, 39]. Pupillometry offers another, converging method, to study this, as well as a means to determine precisely when the stress-buffering effect occurs. A recent theoretical model by Jakubiak and Feeney [74] posits that affectionate touch enacts immediate neurobiological changes, which reduce stress and promote health. The present study is aimed at observing an immediate ANS response shown through pupillary dilations. Additionally, despite the current breadth of work using pupillometry, there is no work that the authors know of that has examined the stress-buffering effects of social support on the pupillary stress response.

Thus, the aim of this innovative study was to examine the effect of received emotional support via hand-holding on the dampening effects of the ANS’s pupillary response. Based on prior research regarding stress response, we expected individuals exposed to the experimental stressor (i.e. Stroop task) would demonstrate increased pupil dilation. Additionally, we expected individuals who receive emotional support, in the form of hand-holding, from their spouse during the stress task would show less pupil dilation in response to the stress task compared to individuals that did not receive emotional support from their spouse.

## Method

### Participants

We predetermined our sample size based on previous pupillometry studies [54, 60, 62]. Thus, 45 couples (N = 90) were recruited from Brigham Young University and the surrounding community. Eligible participants were heterosexual couples, married at least 2 years, who were 21 years of age or older, had normal or corrected-to-normal vision, and had normal color vision. Blood pressure and self-reported stress were analyzed as secondary measures. Demographic information is presented in Table 1. Eligible couples were randomly assigned to the support or non-support condition in order to control for variations in participant variables such as age and length of marriage. In the support condition, couples were assigned to come to the lab together and husbands and wives were randomized to either the participant role first or support-providing role first. One spouse (the participant) completed a stress task while their partner sat across the table providing emotional support by holding the participant’s hand. Spouses providing support were given the following instructions: “Your role as the participant’s spouse is to be engaged in providing support via hand-holding and to avoid activities that would interfere or distract with the provision of that support such as playing on the phone or reading.” Following completion of the task, the spouses changed places and the prior support-providing partner (now the participant) completed the stress task, while their spouse held their hand. Examination of objective stress measures (i.e., pupil dilation and blood pressure) found no differences between role assignment (i.e., support-providing or participant). In the non-support condition, husbands and wives were randomly assigned to participate in either session one or session two. One spouse arrived at the lab and completed the stress task (session one). Within 72 hours (session two), the second spouse came to the lab and completed the stress task. The first spouse was instructed not to discuss the study task details with their partner until both had completed the study, which we verified with the session two participant. Examination of objective stress measures (i.e., pupil dilation and blood pressure) also found no differences between session assignment. Blood pressure measures were taken at baseline, during the task, and during the recovery time following the task. This study was approved by the Brigham Young University Institutional Review Board.

**Table 1:** Demographic information.

	Mean (*SD*)	Range	N	%
Age in years	31.28 (10.6)	21–74		
Marriage length (years)	7.56 (8.98)	2–45		
BMI	25.30 (4.29)	16.14–34.74		
Ethnicity: White			78	87.64
Education status: Some college education			73	82.02
Income: > 50,000			38	42.70
Self-reported health: ≥ Good health			78	87.64

### Procedures

Following consent, participants rested for five minutes and then were fitted with a blood pressure cuff. Three baseline readings were obtained, each one minute apart. Height and weight were collected and the participant completed pre-assessment surveys (for a detailed assessment of these surveys, see Measures, below). Following this, the participant was positioned in front of the eye tracker. In the support condition, the partner of the participant was consented and positioned across the table, unable to see the computer screen.

In addition to hand-holding being an effective form of social support in reducing stress [32, 33], we used this method as the emotional support behavior as it is a ubiquitous method of touch communication and a common way that married couples express support [75]. Additionally, it conveys clear, non-verbal communication of comfort and security [34]. Couples were instructed to hold hands throughout the duration of the task. Both the participant and the partner wore noise-reducing headphones; the participant’s was a headset with mic used for voice onset time and the partner listened to background music. Couples then changed places; the support-giving partner became the participant, and the participant became the support-giving partner. Both were consented in their new roles and the study proceeded again as outlined above. In the non-support condition, no partner was present and the participant simply placed their hands on the table in front of them.

The Stroop task, controlled by E-Prime software, consisted of 84 trials of each stimulus type (control, congruent, or incongruent words; see description below) which were randomized and presented one-at-a-time for 2 seconds each. Participants were to name the color of the font presented. Between each stimulus, a string of hash marks (####) was presented for 1 second. Our procedures were modeled after those outlined by Laeng et al [59]. Voice onset time, response accuracy, and pupil dilation were measured during the Stroop task. Participant’s blood pressure was measured before the task, halfway through the task, and immediately following completion. To account for potential measurement confounds from the blood pressure cuff inflation, we did not record voice onset time, response accuracy, or pupil dilation during the mid-task blood pressure reading. Additionally, because the pupillary light reflex is the main potential confound in cognitive pupillometry [58], the luminance of every aspect of our experimental conditions were controlled for (laboratory, stimuli, inter-stimuli luminance). Participants completed the remainder of the surveys following their own stress task.

### Measures

A demographic questionnaire assessed standard variables including age, income, education, and occupational status.

#### Pupillometry tasks

Pupil dilation and constriction data was acquired using the Tobii TX300 eye-tracking system, controlled by E-Prime software and the E-Prime extensions for Tobii. A low-intensity infrared light is reflected off the participant's retina, and infrared cameras then identify the pupils in each eye. During the task the camera recorded the size of the pupils, as well as where on the computer screen participants directed their attention. Eye position and pupil size were recorded at 250Hz. Preceding the Stroop module and again half-way through, the Tobii would complete a nine-point calibration test to ensure pupil measurement. Each calibration lasted about 20s. The Stroop module lasted for 13 minutes and pupil data was recorded throughout. The presentation of each Stroop stimulus would not occur until the participant had been fixating on the hash mark string (####) in the center of the screen for 500ms. If the participant’s gaze was not within the defined region, the trial would pause until they were within range. This ensured that participants were fixated on the target stimulus when it appeared. Pupil size data was lost during blinks, when the participant looked away from the center of the screen, or during track loss due to poor calibration. Participants with more than 25% pupil size data loss were excluded from pupillary analysis. One participant in the non-support condition was also excluded when the spouse failed to arrive for their portion of the study. In total, eleven participants were excluded.

Lab assessed height and weight were used to calculate BMI and used to control for blood pressure as a BMI below 18.5 and above 29.9 significantly impact blood pressure [76, 77]. A Dinamap Model 100 Pro monitor was used to measure systolic blood pressure (SBP), diastolic blood pressure (DBP), and pulse rate (PR). Assessments were obtained via a properly sized occluding cuff positioned on the non-dominant upper arm. Each participant rested for five minutes before the three baseline blood pressure readings, which were each spaced one minute apart. These were averaged together to create a baseline to increase reliability [78]. Cardiovascular measures were calculated by baseline to mid-task for SBP, DBP, and PR.

#### Spielberger state-trait anxiety scale (STAI)

This measure was given to participants both before and following the pupillometry tasks. Participants rated their current feelings on a 1 (not at all) to 4 (very much) point scale. In our study, the STAI evidenced good internal consistency (Cronbach's α > .76). This is similar to prior work with the STAI [79].

#### The perceived threat and perceived challenge scale

This measure [80] was used to assess participants’ appraisal of the study tasks. Participants were asked a single question, pre and post task, regarding their feelings of both threat and ability to cope with the task. These two questions were rated on a 1 = not at all and 6 = very much Likert scale. These appraisal variables are associated with multiple stress theories which refer to stress as a relative balance between demands and resources [81, 82].

#### Perceptions of control scale

With this measure [83] participants were asked to rate the amount of control they perceived they had over the evaluation of the task on a 1–10 point scale with 1 = low and 10 = high.

## Results

### Data analysis

Behavioral data was analyzed in Stata, version 15 [84] using repeated measures ANOVA for all baseline and mid/post-task variables including cardiovascular measures, Stroop task behavior, and self-reported questionnaires. Pupillary data was analyzed using linear mixed-effects models [85] in R [86]. All models included nested random by-participant and by-couple intercepts and random slopes for all within-participant variables, except when these random slopes prevented model convergence. Field-standard *p*-values (*p* < .05) and confidence intervals (95%) determined significance levels. These *p-*values were obtained using the *lmerTest* package [87] by applying the Satterthwaite approximation for degrees of freedom [88]. For each model, the final random effects structure is specified (see below). For the Stroop task, behavioral responses were analyzed for color naming response time and accuracy. Pupil size was analyzed in two ways, tonic and phasic [89]. Tonic pupil size reflects general arousal, and longer-term changes in that arousal. This measure therefore reflects habituation to the task over time. The phasic pupillary response represents changes in pupil size (and thus arousal) time-locked to the onset of a stimulus (in this case, individual Stroop words). This analysis was conducted to determine if pupillary responses to these immediate stressors were dampened by the support of the spouse. With each stimulus, the pupils dilate and reach two peak diameters. The first peak represents a pre-cognitive pupillary response to the onset of the visual stimulus (i.e. the appearance of the Stroop stimulus on the screen). The second peak is evaluative to the meaning/content of the stimulus. These second pupillary responses are where differences between the support and non-support conditions would appear if stress-buffering was occurring. Thus, the phasic pupillary data was analyzed using a quadratic function beginning at 750ms after each trial onset in order to accurately represent cognitive arousal/stress. In sum, tonic pupil size reflects arousal changes in response to the task, while phasic pupil response reflects changes in pupil size in response to a particular stimulus/trial. For all analyses, predictor variables included Stimulus Type (non-word, congruent word, or incongruent word) and Social Support Condition (support vs. non-support).

### Supplementary analyses

Two-way ANOVA with one repeated measure (Time) were conducted to compare baseline SBP, DBP, and PR with task SBP, DBP, and PR. Collapsing across conditions, there was a significant difference in baseline SPB (*M* = 119.42, *SD* = 10.98) and task SBP (M = 122.91, *SD* = 12.40; *F*(1, 87) = 25.90, p < .001, η^2^ = .23). DBP and PR yielded similar outcomes (see Table 2 for cardiovascular and self-report results). These results are consistent with prior research [70] and indicate a cardiovascular stress response. Additionally, self-report measures indicated that participants found the task stressful. We further examined cardiovascular reactivity differences between the support and non-support groups using repeated measures ANOVA. We found no differences between support conditions (*F*(1, 87) = .471, *p* < .495, η^2^ = .005) indicating that both groups were equally impacted by the stress task.

**Table 2:** Descriptive and ANOVA tests for supplementary measures.

	Pre-task		Mid-task		Post-task						
	*M*	*SD*	*M*	*SD*	*M*	*SD*	*Df*	MS	*F*	η^2^	*p*
Systolic blood pressure	119.42	10.98	122.91	12.40			1, 87	537.42	25.90	.23	.001***
Diastolic blood pressure	72.20	8.87	75.89	9.05			1, 87	606.02	50.50	.37	.001***
Pulse rate	74.04	11.67	77.29	12.09			1, 87	467.16	33.12	.28	.001***
Perceptions of control scale	5.98	2.67			5.85	3.04	1, 87	.66	0.20	.00	.656
Spielberger state-trait anxiety scale	18.25	3.95			21.01	6.50	1, 87	343.51	21.85	.20	.001***
Perceived threat scale	1.40	0.65			1.60	0.94	1, 87	2.03	4.37	.05	.039*
Perceived challenge scale	5.56	0.89			5.36	1.11	1, 87	1.79	3.16	.04	.080

*M* = Mean; *SD* = Standard Deviation. Perceptions of control scale ranges from 1–10, higher scores indicating more control. Spielberger state-trait anxiety scale ranges from 13–52, higher scores indicating higher anxiety. Perceived threat and challenge scales range from 1–6, higher scores indicating higher threat/challenge.

*** *p* < .001.

** *p* < .01.

* *p* < .05.

Supplementary analyses also included evaluation of accuracy and response times to the Stroop task. As expected, error rates for the Stroop task were relatively low and consistent among all participants (*M*_error_ = 1.12, *SD* = 1.73) with 87.76% recording two errors or less. Other studies involving the Stroop task report similar error rates [90, 91]. Response time to the Stroop conditions (i.e. control, congruent, and incongruent) in milliseconds were calculated across all participants and trials. Repeated measures ANOVA revealed that all participants took longer to respond to the incongruent trials than the congruent trials (*F*(2, 166) = 175.5, *p* < .001, η^2^ = .68). These results are consistent with prior research [73, 90]. This analysis also revealed that there was no significant differences between support conditions (*F*(2, 166) = .073, p = .93, η^2^ = .001). This suggests that cognitive load on the Stroop task was not influenced by the emotional support from their spouse. In all, these supplementary behavioral data suggest that participants in this study behaved as expected during the experiment.

We also examined associations between pupillometry, cardiovascular, and self-report data. These analyses did not reveal statistically significant correlations (see supplementary material S1 Table).

### Tonic pupil response

Tonic pupil size was defined as the average pupil diameter during the interval when there was no stimulus presented (i.e. prior to each Stroop trial). For this analysis, the mean pupil size during the interval prior to each trial was computed. During this time, fixation hash marks were presented in the center of the screen, and the program required participants to fixate on these marks for at least 500ms before the next trial would begin. Pupil size measures from these inter-trial intervals were analyzed as a function of Spouse Condition (Support vs. Non-Support) and of Trial, to see if tonic pupil size changed across trials and if this change was moderated by the emotional support from the spouse. The final model included nested random intercepts for participant and for couple.

**Fig 1:**
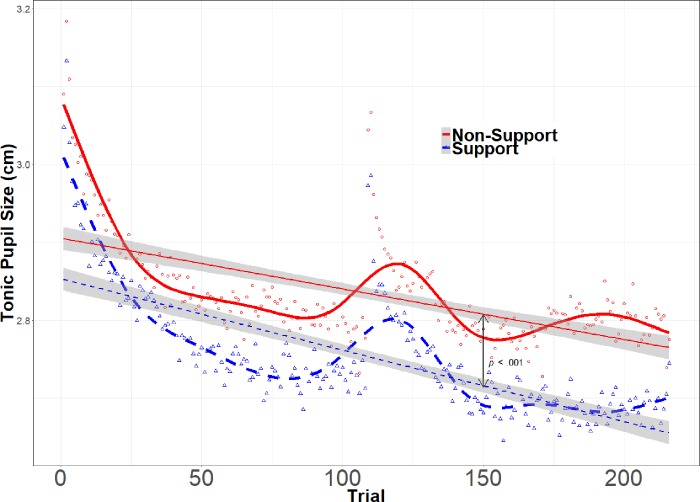
Change in tonic pupil size as a function of trial. Graph shows pupil size change in centimeters across all Stroop trials. The thicker, curved lines represent the raw data. The thinner, straight lines represent the linear function fit by the model. The small red circles and blue triangles represent the averaged data points for each trial by condition.

The tonic analysis revealed that there was no statistical difference in average pupil diameter between support conditions at the beginning of the experiment; all participants started at relatively the same baseline diameter (see Table 3). There was a significant effect of Trial, indicating that tonic pupil size decreased during the course of the experiment. This effect interacted with Spouse Condition, indicating that the effect was stronger in the support condition (*b* = -0.0002, SE = .000035, *t* = -5.76, *p* < .001). In other words, participants who received emotional support from their spouses showed an accelerated habituation to the Stroop task, as measured by tonic pupil size (see Fig 1).

**Table 3:** Model output of the analysis of tonic pupillary response.

Fixed effects	*b*	*SE*	*t*	*p*
(Intercept)	2.82	.0051	55.16	.001***
Trial	-0.00075	.000025	-30.59	.001***
Spouse Condition = Support	-0.072	.074	-0.99	.33
Interaction: Trial X Spouse Condition = Support	-0.0002	.000035	-5.76	.001***

Pupil size decrease during the course of the experiment. There was not a significant difference between the two groups for their intercepts but there was an interaction of trial and support indicating that pupil size decreased faster for the support condition.

*** *p <* .001.

### Phasic pupil response

**Fig 2:**
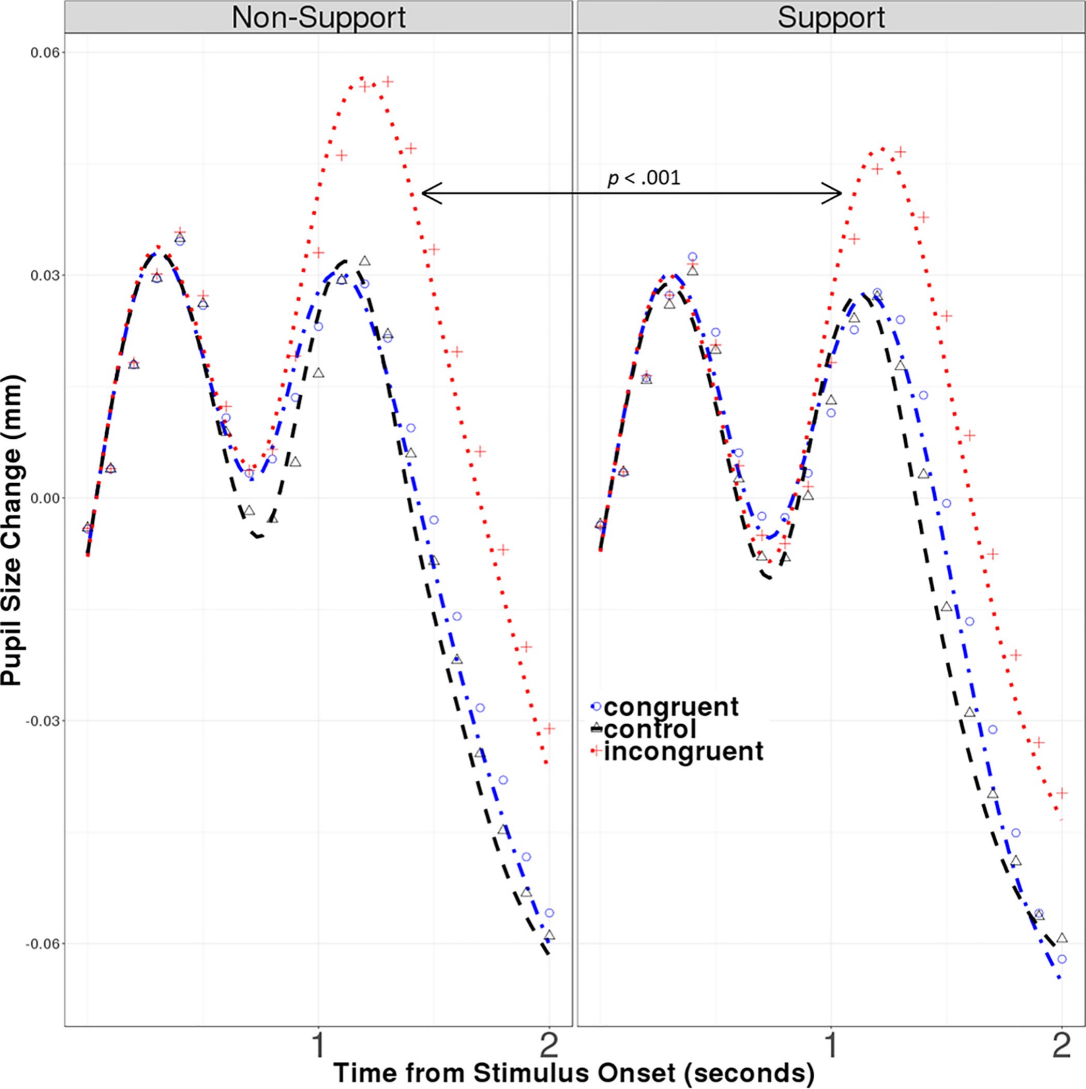
Graph of phasic pupil dilation change represented by raw data. Average pupil dilation change as a response to each individual Stroop task trial shown in millimeters. The data points are binned into 100ms and then averaged for all participants. The graph depicts both support and non-support conditions. The first dilation peak in the fig (from about 0ms - 750ms) represents a reflexive pupillary response to the onset of the visual stimulus (i.e. the appearance of the Stroop stimulus on the screen).The second dilation peak (from 750ms– 2000ms) represents task-specific arousal associated with semantic/evaluative processing of the stimulus. Only the second peak was of interest, so only pupillary data after 750ms post stimulus onset was included in the analysis reported in Table 2.

In the phasic pupil analysis, we assessed the acute physiological reaction to the individual Stroop trials via pupil dilation. In order to model the phasic pupil response to the individual Stroop stimuli, growth curve analysis was employed [92]. This analysis fits a quadratic function beginning at 750ms after stimulus onset to the pupil size data to capture the change in pupil size over time. Fig 2 depicts the pupillary data. Statistical interactions between this Time function and the Stroop Condition (Control, Congruent and Incongruent) reveal differences in the pupillary response to the different types of Stroop stimuli. The critical test of our hypothesis is a three-way interaction of Time, Stroop Condition, and Support Condition, which would indicate that the difference in pupillary response between the congruent and incongruent Stroop conditions was moderated by the received emotional support from the spouse. In addition to these fixed effects and interactions, the final model also included nested random intercepts for participant and for couple and random by-participants slopes for Stroop Condition.

In this analysis, pupil size change was the dependent variable, rather than raw pupil size. This transformation was accomplished by subtracting the raw pupil size at trial time 0 from raw pupil size at each time point. Analyzing pupil change from the pre-trial baseline is standard practice when studying phasic pupil response to a particular stimulus [59], and the tonic pupil analysis revealed that it was a necessary step in order to control for differences across groups and trials (see above). The amount of pupil change observed was consistent with other pupillometry studies [58, 93]. The results of the phasic analysis are shown in Table 4 and Fig 2. This analysis revealed a significant interaction of Time and Stroop Condition (Incongruent), indicating a greater pupillary response in the incongruent condition (compared to the congruent condition) for the non-support group. A significant three-way interaction of Time, Stroop Condition (Incongruent) and Support Condition indicated that the effect of the incongruent condition, while still significant, was smaller for the support group (*b* = 3.89, SE = .60, *t* = 6.48, *p* < .001). In other words, when participants received emotional support from their spouse, their phasic pupillary response to the incongruent stimuli was reduced.

**Table 4:** Model output of the analysis of phasic pupillary response.

Fixed effects	*b*	*SE*	*t*	*p*
(Intercept)	-0.0044	.0078	-0.56	.58
Linear Time Function	-54.70	.29	-186.33	.001***
Quadratic Time Function	-23.40	.29	-79.74	.001***
Stroop = Control	-0.0043	.0032	-1.33	.19
Stroop = Incongruent	0.025	.0038	6.49	.001***
Spouse Condition = Support	-0.0039	.011	-0.34	.73
Linear Time Function X Stroop = Control	1.03	.41	2.48	.01*
Quadratic Time Function X Stroop = Control	-2.60	.42	-6.27	.001***
Linear Time Function X Stroop = Incongruent	15.50	.42	37.40	.001***
Quadratic Time Function X Stroop = Incongruent	-16.40	.42	-39.60	.001***
Linear Time Function X Spouse Condition = Support	-0.64	.43	-1.50	.14
Quadratic Time Function X Spouse Condition = Support	-8.77	.42	-20.66	.001***
Stroop = Control X Spouse Condition = Support	-0.00071	.0046	-0.16	.88
Stroop = Incongruent X Spouse Condition = Support	-0.0067	.0055	-1.22	.23
Linear Time Function X Stroop Control X Spouse Condition = Support	-2.26	.60	-3.77	.001***
Quadratic Time Function X Stroop Control X Spouse Condition = Support	10.30	.60	17.25	.001***
Linear Time Function X Stroop Incongruent X Spouse Condition = Support	-0.018	.60	-0.03	.98
Quadratic Time Function X Stroop Incongruent X Spouse Condition = Support	3.89	.60	6.48	.001***

For the dummy coded Stroop variable, the comparison category was the congruent condition. For the Spouse Condition variable, the comparison category was the Non-Support Condition.

*** *p* < .001.

** *p <* .01.

* *p* < .05.

## Discussion

The purpose of the current study was to examine the stress-buffering effect of received emotional social support on the ANS’s pupillary response. This method provided real-time feedback on the body’s physiological stress response. Additionally, it frames a potentially fruitful method of further investigation in understanding the causal mechanisms linking relationships and health. This novel experiment manipulated received spousal support to explore the dampening effect of emotional support in the form of hand-holding on physiological stress response. We found significant differences between the two manipulated conditions of support and non-support in both tonic and phasic pupillary response. Social Baseline Theory posits that the presence of a spouse would result in less psychological and physiological arousal compared to being alone. Additionally, the stress-buffering hypothesis asserts that social relationships are beneficial in reducing stress-evoked reactivity during periods of acute stress. The results from the current study are consistent with the theories that, being in the presence of one’s spouse may dampen the physiological stress-evoked response, and this ANS dampening can be observed via pupil dilation; participants who received emotional support from their spouse by holding their hand had less pupil dilation in response to the Stroop task. These results extend the current literature by using a novel method to investigate these associations, and provide additional insights into the speed at which these effects happen and can be captured.

To date, pupillometry has been a validated form of measuring ANS activity and stress [54, 60–65]. The practical significance of pupillometry is that it is another unobtrusively applied method in stress detection. It can be conducted via video cameras [94] and used in various research designs. In addition to the practical application of pupillometry, these results add to basic science. In line with previous studies, we found novel, converging evidence, that receiving emotional support from a significant other can attenuate stress reactivity during an acute stressor. This collective finding from diverse measures further validates these results as robust effects. In accordance with other validated physiological markers of stress, each method converges to broaden our understanding of the potential salubrious nature of social support. Pupillometry adds the unique component of detecting the influence of social support on stress within milliseconds.

The tonic pupil analysis revealed that average pupil size decreased during the course of the Stroop task, such that the pupil was more dilated at the beginning than at the end of the experiment, indicating a desensitization to the task. Such desensitization is justified; a person exposed to repeated identical situations may re-evaluate a stressful circumstance as being more neutral [95, 96]. Additionally, this provides further evidence of unconscious physiological desensitization, as one cannot consciously control pupil dilation or constriction [97]. This neutralizing effect interacted with social support, indicating that the effect was stronger in the support condition. In other words, participants who received emotional support from their spouse in the form of hand-holding showed an accelerated habituation to the Stroop task. The implications of these findings support the stress-buffering hypothesis that receiving support from a spouse is beneficial during stressful events.

From the phasic pupil analysis, we were able to confirm that, as expected, there was no difference in pupil response between congruent and control Stroop trials. However, there was a significant difference between these trials and the incongruent Stroop trials. This demonstrates that the Stroop task was stressful and indeed, provided differential pupil effects [59]. Additionally, there was a difference in the size of the inconguency effect between experimental conditions: Those who received support during the Stroop task had less pupil reactivity to the incongruent trials than those who were alone. This suggests that, in accordance with previous social support research, emotional support from a spouse during exposure to a stressor can dampen physiological reactions. In addition to other well-established biomarkers of physiological stress-response (e.g. cardiovascular, immune, skin conductance, and hormones) these results provide evidence that pupil dilation is sensitive to the effects of stress-buffering. This finding also reveals the speed at which stress-buffering occurs, suggesting that the dampening effects of social support are immediate. Future research should address applications of this method to clinical and intervention work. Specifically, phasic pupil analyses allow for immediate, precise, and time dependent detection of acute stress (and stress-buffering), thus allowing researchers to parcel out various stimuli and their potential independent effects. For example, during a couple’s conflict or stress task, the effect of various reactions, stressors, tasks, or support giving/support receipt may not be equally impactful. Because pupil dilations are immediate and recovery time between each stimuli is merely seconds, each of these different aspects could be segmented and their independent effects analyzed. This could allow the clinician to discern specific problem areas within the relationship and create a personalized plan of action for the couple based on their reactions to specific stimuli. This enables clinician objectivity and precision. Similarly, this allows for the investigation of multiple behaviors of interest in a single session.

A recent article by Jakubiak and Feeney [74] proposes a theoretical link as to how touch can be health promoting. These authors argue that affectionate touch promotes relational, psychological, and physical well-being. Indeed, a proposed pathway by which their model posits touch affects well-being is by immediate neurobiological changes, which reduce stress. According to Jakubiak and Feeney, the primary future research need in this area is to investigate these pathways experimentally. From the present study’s results, the reduction of stress reactivity shown through the ANS’s pupillary response happens almost immediately, lending experimental evidence to theorized assumptions that supportive physical touch is health promoting. Pupil dilations in response to stress are caused by the LC. Increased secretions of norepinephrine by the LC has been theorized to be a major contributing factor in modulating activity of the HPA axis [98, 99]. Pupillometry is a method to investigate this important neurological pathway in the chain of autonomic stress response and subsequent health outcomes. Future research should combine methods to directly test the pupillary-neuronal correlations in response to stress.

Overall, these findings support prior research that physical touch from a supportive spouse during situations of acute stress can attenuate physiological reactivity. While our findings indicate that support from a spouse can attenuate the immediate physiological stress reaction (phasic) and help habituate the individual to the stressful situation faster (tonic), we cannot determine if this habituation would extend to longer term stressors. Future research is needed, including intervention work, to confirm these findings on various situations of acute stress such as daily hassles, or relationship conflict.

Interestingly, the correlational analyses of pupillary and cardiovascular data did not yield significant results. The lack of cardiovascular effects between the support and non-support conditions could possibly be addressed by the study design. This was an exploratory study aimed at the effects of pupillary changes. Pupillary measures were recorded every 250Hz for each of the 90 participants, resulting in over 20 million data points. This provided high precision for the pupillary analyses. In contrast, cardiovascular measures were a supplementary analysis aimed at assessing acute stress reactivity generally, with only a few readings. Thus, the lack of cardiovascular effects between the two support conditions is somewhat unsurprising given the study methods. Additionally, Ditzen and Heinrichs [9] explain that autonomic activation can be assessed through different physiological markers, such as pulse rate, blood pressure, or skin conductance, but that these measures do not necessarily correlate. This could be due to the different temporal sensitivity of the different methods. It also could be that different methods capture activation of different neurological sub-systems.

There are several potential reasons why there were not significant correlations between self-report and objective measures. Kuchinsky et al [97] mentions two possible explanations. The first is that people vary in their ability to introspect and be unbiased in their responses. Secondly, surveys are filled out after task completion, thus relying on memory and summation so self-report responses could reflect an average of perceived stress across many trials and many different conditions. Thus, those with the most physiological reactivity may not accurately recall themselves as being stressed or vice versa.

Cognitive load can enlarge pupil dilation [100–102]. However, if the effects observed were cognitive load and not stress, we would not expect to see differences between support and non-support conditions in pupil dilation. Additionally, we would expect to see differences between the two groups’ response times and accuracy rates to the incongruent trials. That is, those in the support condition would have faster response times and less errors to the incongruent trials of the Stroop task compared to those in the non-support condition. However, response time and accuracy analysis revealed that both groups performed similarly on the task. This provides additional evidence that the pupil dilation difference between the two groups is not due to cognitive load. In line with this, our ancillary analysis revealed that participants perceived the Stroop task as stressful and that there was a statistically significant cardiovascular response. In all, this suggests that the pupil change is the result of a stress response and not cognitive load.

There are several methodological limitations to this study. As the support condition manipulated both spousal presence and touch simultaneously, it is possible that the effects seen are elicited only through the physical sensation of spousal touch or only by the presence of a spouse, or, perhaps both are necessary contributing factors. Future studies could examine these independently. Similarly, although hand holding has been shown to be an effective form of received emotional support [32–34], we did not explicitly ask participants in the support condition if they *felt* this hand-holding from their spouse to be supportive. The present study aims were addressed toward received support only, however, perceived support has been shown in the literature to be an important aspect in stress-buffering. Future studies should extend these findings in an investigation of perceived support.

Because this was an exploratory study and, to our knowledge, the first study aimed at examining stress-buffering effects of social support on pupillary stress response, the use of pupillometry to assess stress response is novel. More research is needed to confirm and extend our findings. The Stroop task has been shown in the literature to be physiologically and psychologically stressful, and self-reported behavioral measures indicate that participants found the task marginally stressful, but future work could include a more stressful task. This may yield even greater physiological reactivity and stronger effects of stress-buffering. Increasing the stress effect possibly would have yielded significant differences between the support conditions on cardiovascular and self-report variables. Additionally, the present study design was limited in sample size and thus unable to address the more complex research question of the moderating effects of relationship quality. From the literature, it is clear that perceived relationship quality is an important factor in stress-buffering and health benefits. This body of research posits that in addition to marital status, which offers stress-buffering benefits, the *quality* of the marriage provides additional and often greater effects associated with stress-buffering and health outcomes [39, 103, 104]. Future studies should include larger sample sizes that could examine this important aspect of stress-buffering. An investigation of different paradigms of social support would be informative as well. For instance, an examination of simply the presence of a partner, or perceptions of a non-present partner’s support would provide additional detail. Further, investigating whether these results would be observed with other support members such as other family, close friends, acquaintances, or even strangers would be important.

The present study adds to previous work showing that spousal emotional support dampens acute stress reaction by employing a novel approach to studying stress-buffering and social support: pupillometry. Additionally, this study contributes to the literature by suggesting that the speed at which stress-buffering occurs is nearly instantaneous. This finding could be important in intervention and clinical applications. More work is needed to investigate this immediate association and its implications. Pupillometry methods add a new, unique, and potentially fruitful tool to the already established measures used to investigate social support.

## Supporting information

S1 Table Blood pressure and self-report correlations. *Note*. Sbp = systolic blood pressure; Dbp = diastolic blood pressure; Hr = pulse rate; STAI = Spielberger state-trait anxiety scale; PT = perceived threat scale; PC = perceived challenge scale; POC = perceptions of control scale. * *p* < .05.
(DOCX)
