# Supplementary material for: Supportive hand-holding attenuates pupillary responses to stress in adult couples
Source: PLoS One. 2019 Feb 22;14(2):e0212703. doi: 10.1371/journal.pone.0212703 (PMC6386442; doi:10.1371/journal.pone.0212703)
Supplement: S1 Table — Note. Sbp = systolic blood pressure; Dbp = diastolic blood pressure; Hr = pulse rate; STAI = Spielberger state-trait anxiety scale; PT = perceived threat scale; PC = perceived challenge scale; POC = perceptions of control scale. * p < .05. (DOCX) [file pone.0212703.s001.docx]

| Table S1 |  |  |  |  |  |  |  |  |  |  |  |  |  |  |
| --- | --- | --- | --- | --- | --- | --- | --- | --- | --- | --- | --- | --- | --- | --- |
| *Blood Pressure and Self-Report Correlations* | | | | | | | |  |  |  |  |  |  |  |
| Variable | 1 | 2 | 3 | 4 | 5 | 6 | 7 | 8 | 9 | 10 | 11 | 12 | 13 | 14 |
| 1. Sbp_pre-task | - |  |  |  |  |  |  |  |  |  |  |  |  |  |
| 1. Dbp_pre-task | 0.77* | - |  |  |  |  |  |  |  |  |  |  |  |  |
| 1. Hr_pre-task | 0.14 | 0.16 | - |  |  |  |  |  |  |  |  |  |  |  |
| 1. Sbp_mid-task | 0.86* | 0.72* | 0.15 | - |  |  |  |  |  |  |  |  |  |  |
| 1. Dbp_mid-task | 0.67* | 0.85* | 0.11 | 0.77* | - |  |  |  |  |  |  |  |  |  |
| 1. Hr_mid-task | 0.12 | 0.17 | 0.90* | 0.16 | 0.16 | - |  |  |  |  |  |  |  |  |
| 1. STAI_post-task | -0.06 | -0.11 | 0.17 | -0.19 | -0.16 | 0.09 | - |  |  |  |  |  |  |  |
| 1. STAI_pre-task | 0.05 | 0.10 | 0.22* | 0.07 | 0.08 | 0.14 | 0.58* | - |  |  |  |  |  |  |
| 1. PT_post-task | -0.08 | -0.02 | 0.03 | -0.13 | -0.07 | -0.05 | 0.55* | 0.43* | - |  |  |  |  |  |
| 1. PT_pre-task | -0.18 | -0.01 | -0.01 | -0.09 | -0.04 | -0.01 | 0.12 | 0.33* | 0.31* | - |  |  |  |  |
| 1. PC_post-task | -0.08 | -0.16 | -0.16 | -0.03 | -0.11 | -0.13 | -0.17 | -0.15 | -0.20 | -0.28* | - |  |  |  |
| 1. PC_pre-task | -0.02 | -0.12 | -0.13 | -0.08 | -0.16 | -0.14 | -0.15 | -0.25* | -0.16 | -0.26* | 0.42* | - |  |  |
| 1. POC_post-task | -0.24* | -0.19 | 0.00 | -0.14 | -0.05 | 0.02 | -0.08 | 0.06 | -0.07 | 0.02 | 0.14 | -0.06 | - |  |
| 1. POC_Pre-task | -0.24* | -0.15 | -0.07 | -0.24* | -0.13 | -0.05 | -0.11 | -0.11 | 0.04 | 0.04 | -0.14 | 0.10 | 0.60* | - |

*Note.* Sbp = systolic blood pressure; Dbp = diastolic blood pressure; Hr = pulse rate; STAI = Spielberger state-trait anxiety scale; PT = perceived threat scale; PC = perceived challenge scale; POC = perceptions of control scale. * *p* < .05.
